# Effectiveness of the 23-Valent Pneumococcal Polysaccharide Vaccine in Reducing Mortality Among Older Adults: A Real-World Retrospective Cohort Study in Guangzhou, China

**DOI:** 10.3390/vaccines14100877

**Published:** 2026-10-02

**Authors:** Yan Kang, Zhiwei Zheng, Juan Qi, Lin Tang, Wen Wang, Xinqi Lin, Qiaojuan Liu, Wenji Wang, Chunhuan Zhang, Jianxiong Xu, Weijia Liu, Zhoubin Zhang, Boheng Liang, Yong Huang

**Affiliations:** 1 Guangzhou Center for Disease Control and Prevention (Guangzhou Health Supervision Institute), Guangzhou 510440, China; kangy_11@126.com (Y.K.); gzcdc_zhengzw@163.com (Z.Z.);; 2 Institute of Public Health, Guangzhou Medical University & Guangzhou Center for Disease Control and Prevention (Guangzhou Health Supervision Institute), Guangzhou 510440, China

**Keywords:** pneumococcal vaccines, elderly, mortality, cohort studies, vaccine effectiveness

## Abstract

**Objectives**: To evaluate the real-world effectiveness of the 23-valent pneumococcal polysaccharide vaccine (PPSV23) in reducing all-cause, pneumonia-related, and chronic obstructive pulmonary disease (COPD)-related mortality in adults aged ≥60 years. **Methods**: In this retrospective cohort study using existing health surveillance data, individuals aged ≥60 years on 1 January 2020 were followed until 31 August 2025 and classified by PPSV23 vaccination status. Propensity score matching (PSM) at a 1:1 ratio balanced sex, age, regional distribution, urbanization level and influenza vaccination status. Time-dependent Cox proportional hazards models estimated hazard ratios (HR) and vaccine effectiveness (VE), defined as (1 − HR) × 100%. Subgroup, negative control (mortality from traffic accidents), and E-value sensitivity analyses were performed. **Results**: After PSM, 453,500 individuals were included, with a mean follow-up of 5.61 years. All-cause mortality was 3.93 per 1000 person-years in vaccinated versus 9.57 in unvaccinated individuals. PPSV23 vaccination reduced all-cause mortality (VE = 43.1%, 95% CI: 41.2–45.0%), pneumonia-related mortality (VE = 46.2%, 95% CI: 39.6–52.1%), and COPD-related mortality (VE = 33.5%, 95% CI: 20.9–44.2%) (all *p* < 0.001). In a cause-specific decomposition, pneumonia- and COPD-related deaths together explained only 14.9% of the absolute reduction in all-cause deaths, whereas circulatory diseases and malignant tumors accounted for 36.8% and 31.9%, respectively. Among those also vaccinated against influenza, VE was 48.0% and 38.2% for pneumonia- and COPD-related mortality, respectively. For COPD-related mortality, the benefit was greater in men (VE = 42.3%) and in those aged ≥70 years (70–79 years, VE = 45.6%; ≥80 years, VE = 52.0%). PPSV23 was not associated with the negative control outcome (*p* = 0.247); E-values (1.98–2.44) indicated robustness to unmeasured confounding. **Conclusions**: PPSV23 vaccination was associated with significantly lower all-cause, pneumonia-related, and COPD-related mortality among elderly individuals aged ≥60 years. However, the observed association with all-cause mortality should be interpreted cautiously. Continued promotion of PPSV23 vaccination and advocacy for combined influenza–pneumococcal vaccination strategies are recommended.

## 1. Introduction

Pneumococcal disease poses a substantial burden on the elderly population globally [1]. *Streptococcus pneumoniae* (Spn) disproportionately affects individuals aged ≥65 years because of immunosenescence and comorbidities [2], especially chronic obstructive pulmonary disease (COPD) [3]. Pneumococcal pneumonia causes an estimated one million deaths annually, with incidence rates in older adults ranging from 24 to 85 per 100,000 [4]. In China, Spn is a leading pathogen of community-acquired pneumonia (CAP) in the elderly, accounting for 28.0% to 71.5% of cases across regions [5]. Given this substantial and growing population—310 million adults aged ≥60 years by the end of 2024 (22.0% of the total population)—prevention of pneumococcal disease is a critical public health priority [6,7].

The 23-valent pneumococcal polysaccharide vaccine (PPSV23) contains purified capsular polysaccharides from 23 pneumococcal serotypes, which together account for 60–85% of pathogenic strains [8]. These capsular polysaccharides are high-molecular-weight carbohydrate polymers composed of repeating oligosaccharide units, whose monosaccharide composition and glycosidic linkage pattern define serotype-specific antigenic epitopes [9]. As T-cell-independent antigens, they cross-link B-cell receptors, eliciting antibody responses—predominantly IgG2 in adults; the resulting anticapsular antibodies clear pneumococci via complement deposition and Fc receptor-mediated opsonophagocytosis, conferring serotype-specific protection [10,11].

Despite this broad serotype coverage, PPSV23 uptake remains low in China, because it is primarily self-funded in most regions [12]. Although multiple studies have shown PPSV23 to be 45–80% effective against invasive pneumococcal disease [13,14,15,16], its protective effect against all-cause mortality, pneumonia-related mortality, and COPD-related mortality remains controversial, and the evidence is inconsistent [14,15,16]. A 2015 large-scale study from Taiwan, China, demonstrated that PPSV23 vaccination in adults aged ≥75 years reduced pneumonia-related and all-cause mortality by >90% [13], and a 2017 review noted a 40% reduction in COPD exacerbation risk with pneumococcal vaccination [17]. In stark contrast, a 2024 meta-analysis of four randomized controlled trials by Shabil et al. found no significant reduction in all-cause mortality (RR = 1.03), although it did show a significant reduction in pneumonia-related mortality (HR = 0.50) [15]. Adding to the controversy, a 2025 meta-analysis by Bulkhi et al., encompassing 35 studies with over 1.65 million vaccinated individuals, reported that although pneumococcal vaccination reduced all-cause hospitalization, it did not demonstrate a statistically significant reduction in either pneumonia-associated or all-cause mortality in pooled analyses [16]. These discrepancies likely stem from differences in study design and in the ability to control for healthy vaccinee bias—a methodological challenge that underscores the need for rigorous real-world evidence [16].

Since 2023, Guangzhou has provided free PPSV23 to residents aged 65 years and older, creating an ideal setting for a large-scale evaluation. We conducted a retrospective cohort study using population-based surveillance data to test the hypothesis that PPSV23 vaccination is associated with lower mortality risk in adults aged ≥60 years, aiming to support evidence-based vaccination policies.

## 2. Materials and Methods

### 2.1. Study Design and Data Sources

This population-based retrospective cohort study used linked data from multiple health surveillance systems in Guangzhou, China. The study period was from 1 January 2020, to 31 August 2025. Demographic characteristics and vaccination records for adults aged ≥60 years were obtained from the Guangdong Vaccine Administration and Management Information System. Mortality data were retrieved from the Guangzhou Mortality Surveillance System.

### 2.2. Study Population

Eligible participants were adults who reached age 60 years by 1 January 2020 (i.e., born on or before 31 December 1959), with complete identification and vaccination records. The lower age limit of 60 years was predefined based on the national definition of older adults in China and the target population of adult pneumococcal vaccination guidelines [18].

Individuals were excluded if they: had received PPSV23 or the 13-valent pneumococcal conjugate vaccine (PCV13) prior to study initiation, had inconsistent records, or were deceased before the study start.

### 2.3. Exposure Definition

Participants were classified into vaccinated and unvaccinated groups based on PPSV23 receipt during follow-up. Consistent with previous studies [19], we considered vaccination to be effective from 14 days post-administration to allow time for protective antibody development. Accordingly, for individuals vaccinated during the study period, exposure status was modeled as time-varying, switching from “unvaccinated” to “vaccinated” from day 15 post-vaccination onward. Vaccine protection was assumed to persist throughout the study period [20,21].

### 2.4. Outcomes

Deaths were coded using the International Classification of Diseases, 10th Revision (ICD-10). For the purposes of this study, deaths were categorized into 11 groups: diseases of the circulatory system; neoplasms; diseases of the respiratory system; external causes; diseases of the digestive system; endocrine, nutritional and metabolic diseases; diseases of the genitourinary system; infectious diseases; diseases of the nervous system; other causes; and alive at the end of follow-up.

The primary outcomes of interest were: (1) all-cause mortality: death from any cause during follow-up; (2) pneumonia-related mortality: death with an underlying cause coded as J12–J18 (pneumonia, including viral, bacterial, and unspecified pathogens); and (3) COPD-related mortality: death with an underlying cause coded as J44 (chronic obstructive pulmonary disease).

### 2.5. Covariates

To account for potential confounding, we collected data on the following covariates:

Sex: Male or female.
Age: Categorized at baseline as 60–69 years, 70–79 years, and ≥80 years.
Regional distribution: Classified as central urban areas (encompassing Haizhu, Yuexiu, Tianhe, Liwan Districts) and non-central urban areas (encompassing Conghua, Zengcheng, Nansha, Huadu, Baiyun, Panyu, Huangpu Districts), based on geographical location and socioeconomic development level.
Urbanization level: Classified by township and subdistrict, according to the location of the vaccination clinic where the participant’s vaccine record was held.
Influenza vaccination status: Recorded during follow-up. Consistent with previous studies [22], influenza vaccination was considered effective from 14 days post-administration, with protection assumed to persist for 6 months. After this period, vaccination status reverted to “unvaccinated.” Influenza vaccination was therefore modeled as a time-varying covariate. Concurrent vaccination was defined as receipt of at least one dose each of influenza vaccine and PPSV23 during the follow-up period.

### 2.6. Statistical Analysis

Continuous variables were presented as mean ± standard deviation (SD), and categorical variables as frequency and percentage (*n*, %). To control for confounding, 1:1 propensity score matching (PSM) was performed to balance baseline covariates between the PPSV23-vaccinated and unvaccinated groups [23]. Propensity scores were estimated using a logistic regression model. Covariates included sex, age, regional distribution, urbanization level, and influenza vaccination status. Matching was performed using nearest-neighbor matching without replacement, with a caliper of 0.1 times the standard deviation of the linear propensity score. Standardized mean differences (SMD) were used to assess covariate balance; an SMD < 0.1 indicated adequate balance between groups.

After PSM, Kaplan–Meier survival curves were used to compare survival probabilities between the two groups. Mortality density was calculated as deaths per 1000 person-years: (number of deaths during follow-up × 1000)/person-years observed. Given the time-varying nature of PPSV23 and influenza vaccination status during follow-up, time-dependent Cox proportional hazards regression models were used, with the unvaccinated group as the reference, to analyze the association between PPSV23 vaccination and the three mortality outcomes. Both univariable and multivariable models were fitted, and the proportional hazards assumption was tested. To account for potential residual confounding after PSM, the multivariable model was adjusted for age, sex, regional distribution, urbanization level, and influenza vaccination status. Hazard ratios (HR) and their 95% confidence intervals (95% CI) were calculated. Vaccine effectiveness (VE) was calculated as (1 − HR) × 100%.

To enhance the robustness of the findings, subgroup analyses were performed based on key covariates. To strengthen the reliability of the results, deaths from traffic accidents (ICD-10 codes V01–V99) were used as a negative control outcome to assess its association with PPSV23 vaccination. Additionally, E-values were calculated for the primary and secondary outcomes to quantify the potential impact of unmeasured confounding on the observed associations [24]. A larger E-value indicates that stronger unmeasured confounding would be needed to explain the observed association. All statistical analyses were performed using R software (version 4.2.0). A two-sided *p* < 0.05 was considered statistically significant.

### 2.7. Ethics Approval

This study was conducted in accordance with the Declaration of Helsinki and approved by the Ethics and Research Committees of Guangzhou Center for Disease Control and Prevention (GZCDC-ECHR-2023P0043; 19 June 2023). Informed consent was waived because all data were de-identified.

## 3. Results

### 3.1. Baseline Characteristics

A total of 653,414 participants were enrolled. After 1:1 PSM, 453,500 participants (matching rate: 69.41%) were included, yielding 226,750 individuals in each of the PPSV23-vaccinated and unvaccinated groups. The mean follow-up duration was 5.61 ± 0.35 years. The PPSV23 vaccines administered were primarily from two manufacturers: Chengdu Institute of Biological Products Co., Ltd. (Chengdu, China; 87.32%) and Yuxi Walvax Biotechnology Co., Ltd. (Yuxi, China; 7.33%). After PSM, SMD for urbanization level was 0.165, while SMDs for all other covariates were <0.1. Overall, 42.35% of participants were male, and the mean age was 68.53 ± 5.81 years; 38.69% resided in central urban areas and 32.44% in rural township. During the study period, the mean number of influenza vaccine doses received was 1.12 ± 0.91, and 72.27% of participants had received at least one influenza vaccination (Table 1).

### 3.2. PPSV23 and Mortality Outcomes

#### 3.2.1. Kaplan–Meier Survival Analysis

**Figure 1:**
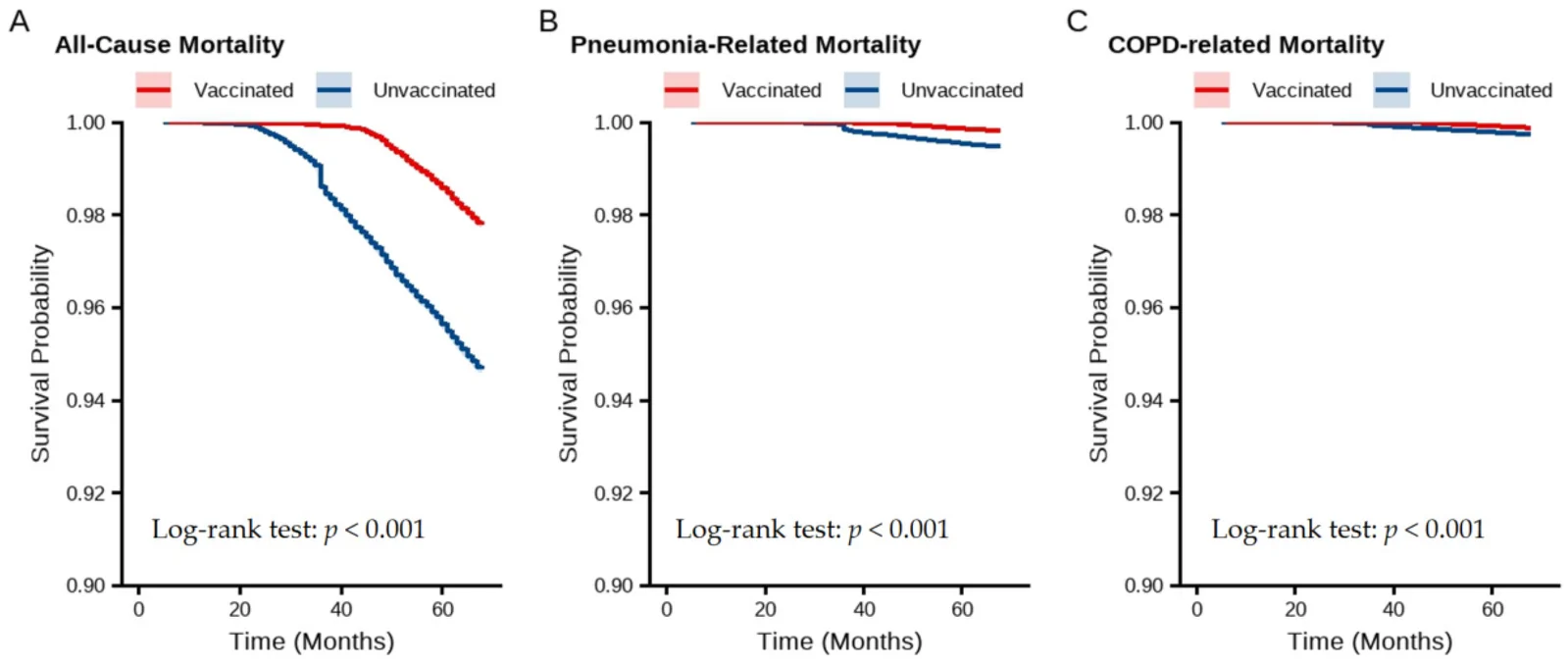
Kaplan–Meier Survival Curves for PPSV23 Vaccination in relation to (**A**) all-cause mortality, (**B**) pneumonia-related mortality, and (**C**) COPD-related Mortality. (Note: The y-axis is truncated at 0.90; absolute mortality is low, so curves appear close).

Kaplan–Meier curves for all-cause, pneumonia-related, and COPD-related mortality are shown in Figure 1A–C. Survival probabilities were significantly higher in the PPSV23-vaccinated group than in the unvaccinated group for all three outcomes (log-rank test, *p* < 0.001 for all). Because mortality was relatively rare, the curves remained close to 1.0, particularly for pneumonia-related and COPD-related mortality; however, the absolute mortality density differences were clinically relevant: 5.64, 0.59, and 0.24 fewer deaths per 1000 person-years for all-cause, pneumonia-related, and COPD-related mortality, respectively. Over a mean follow-up of 5.61 years, this corresponds to approximately 31.6, 3.3, and 1.3 fewer deaths per 1000 vaccinated individuals, respectively.

#### 3.2.2. Association of PPSV23 with All-Cause Mortality

During follow-up, 12,084 all-cause deaths occurred in the unvaccinated group, with a mortality density of 9.57 (95% CI: 9.40–9.74) per 1000 person-years, which was higher than that in the vaccinated group (5033 deaths; mortality density: 3.93 [95% CI: 3.83–4.04] per 1000 person-years). After adjustment for covariates including age, sex, regional distribution, urbanization level, and influenza vaccination, the multivariable time-dependent Cox model revealed that PPSV23 vaccination was associated with a significantly reduced risk of all-cause mortality (HR = 0.57; VE = 43.1%; 95% CI: 41.2–45.0%; *p* < 0.001) (Table 2).

In a descriptive decomposition of the absolute reduction in all-cause deaths in the propensity-score-matched cohort, pneumonia-related and COPD-related deaths together represented 1048 fewer deaths, accounting for 14.9% of the 7051 fewer all-cause deaths. Circulatory system diseases (2595 fewer deaths; 36.8%) and malignant tumors (2246 fewer deaths; 31.9%) accounted for the largest proportions. Within circulatory system diseases, coronary heart disease (1113 fewer deaths; 15.8%) and cerebrovascular disease (1020 fewer deaths; 14.5%) were the main contributors. Within malignant tumors, digestive system tumors (842 fewer deaths; 11.9%) and respiratory system tumors (700 fewer deaths; 9.9%) were the most common. The remaining reductions were distributed across external causes (392 fewer deaths; 5.6%) and the remaining causes combined (Table 1 and Table 2).

Subgroup analyses showed no significant interactions between any covariates and PPSV23 vaccination. Consistent and statistically significant results were observed across all subgroups (Table 3).

#### 3.2.3. Association of PPSV23 with Pneumonia-Related Mortality

The pneumonia-related mortality density was 0.93 (95% CI: 0.88–0.98) per 1000 person-years in the unvaccinated group, which was higher than 0.34 (95% CI: 0.31–0.37) per 1000 person-years in the PPSV23-vaccinated group. In the multivariable time-dependent Cox model, PPSV23 vaccination was associated with a significantly reduced risk of pneumonia-related mortality (HR = 0.54; VE = 46.2%; 95% CI: 39.6–52.1%; *p* < 0.001). Subgroup analyses revealed no significant interactions between any covariates and PPSV23 vaccination. Consistent and statistically significant results were observed across all subgroups, except among individuals without influenza vaccination (*p* = 0.097) (Table 3).

#### 3.2.4. Association of PPSV23 with COPD-Related Mortality

The COPD-related mortality density was 0.46 (95% CI: 0.43–0.50) per 1000 person-years in the unvaccinated group, higher than 0.22 (95% CI: 0.19–0.25) per 1000 person-years in the PPSV23-vaccinated group. In the multivariable time-dependent Cox model, PPSV23 vaccination was associated with a significantly reduced risk of COPD-related mortality (HR = 0.66; VE = 33.5%; 95% CI: 20.9–44.2%; *p* < 0.001). Subgroup analyses revealed significant interactions between PPSV23 vaccination and both age (*p* = 0.023) and sex (*p* = 0.007). In age-stratified analyses, PPSV23 significantly reduced COPD-related mortality among individuals aged 70–79 years (VE = 45.6%; *p* = 0.003) and those aged ≥80 years (VE = 52.0%; *p* = 0.003), whereas the effect did not reach statistical significance among those aged 60–69 years (VE = 28.8%; *p* = 0.059). In sex-stratified analyses, male vaccine recipients derived significant benefit (VE = 42.3%; *p* < 0.001), whereas no significant association was observed among females (*p* = 0.539). Furthermore, stratified analyses indicated that the protective effect of PPSV23 was more pronounced among residents of non-central districts (VE = 42.9%; *p* < 0.001) and those with prior influenza vaccination (VE = 38.2%; *p* < 0.001).

### 3.3. Sensitivity Analysis

As shown in Table 4, PPSV23 was not statistically associated with the negative control outcome (death from traffic accidents; adj HR = 0.79, *p* = 0.247, E-value = 1.64). The E-values for PPSV23 across different outcome variables in Table 2 ranged from 1.98 to 2.44, indicating that the unmeasured confounding of moderate strength would be required to explain the observed associations, although residual confounding cannot be excluded.

## 4. Discussion

This population-based retrospective cohort study of 453,500 propensity-score-matched adults aged ≥60 years in Guangzhou found that PPSV23 vaccination was associated with significantly lower risks of all-cause, pneumonia-related, and COPD-related mortality over a mean follow-up of 5.61 years. In absolute terms, vaccination corresponded to 5.64, 0.59, and 0.24 fewer all-cause, pneumonia-related, and COPD-related deaths per 1000 person-years, respectively. These findings support our hypothesis and provide scarce China-specific evidence that PPSV23 may reduce clinically meaningful mortality under routine programmatic conditions, consistent with the biologic rationale for pneumococcal prevention in older adults, whose risk is amplified by immunosenescence, multimorbidity, frailty, and chronic respiratory disease [1,25,26,27,28].

The 46.2% reduction in pneumonia-related mortality closely mirrors the 2024 meta-analysis by Shabil et al. [15] and aligns with the 2025 meta-analysis showing lower pneumonia-related hospitalization and mortality [16]. Our findings also align with the Japanese VENUS matched cohort, which reported lower pneumonia and all-cause mortality after PPSV23 among adults aged ≥65 years [20], and with hospital-based evidence that prior pneumococcal vaccination is associated with improved short-term survival in elderly patients hospitalized for community-acquired pneumonia [29]. Several design elements strengthen causal inference: PPSV23 and influenza vaccination were modeled as time-varying exposures, reducing immortal-time bias; propensity-score matching and multivariable adjustment improved comparability; and the null association with traffic-accident mortality (*p* = 0.247) argues against a strong generic healthy-user artifact. Although the Kaplan–Meier curves appeared visually close, this reflects the low absolute event rates and the truncated y-axis rather than an absence of effect, as supported by the significant hazard ratios and absolute mortality density differences.

By contrast, the all-cause mortality association in our cohort (VE = 43.1%) should be interpreted more cautiously. In the matched cohort, pneumonia-related and COPD-related deaths together accounted for only 14.9% of the absolute reduction in all-cause deaths (1048 of 7051 fewer deaths), whereas circulatory system diseases (36.8%) and malignant tumors (31.9%) accounted for the largest proportions. Within circulatory system diseases, coronary heart disease (1113 fewer deaths; 15.8%) and cerebrovascular disease (1020 fewer deaths; 14.5%) were the main contributors; within malignant tumors, digestive system tumors (842 fewer deaths; 11.9%) and respiratory system tumors (700 fewer deaths; 9.9%) were most common. These findings are inconsistent with meta-analytic evidence showing that PPSV23 has not consistently reduced all-cause mortality. In a 2024 meta-analysis of four RCTs, Shabil et al. found no reduction in all-cause mortality (RR = 1.03, 95% CI: 0.95–1.12), although pneumonia-related mortality was significantly reduced (HR = 0.50, 95% CI: 0.32–0.69) [15]. Similarly, a 2025 meta-analysis by Bulkhi et al., including 35 studies and more than 1.65 million vaccinated individuals, found no significant reduction in all-cause mortality (OR = 0.71, 95% CI: 0.25–1.98) or pneumonia-associated mortality (OR = 0.69, 95% CI: 0.35–1.39) with PPSV23 alone, despite a modest reduction in all-cause hospitalization [16].

The reduction in circulatory deaths may partly reflect prevention of infection-triggered cardiovascular events, consistent with a meta-analysis of 15 studies including 347,444 participants, which found that pneumococcal vaccination was associated with a lower risk of myocardial infarction (HR = 0.73, 95% CI: 0.50–0.96), although cardiovascular mortality (HR = 0.87, 95% CI: 0.72–1.07) and stroke (HR = 1.01, 95% CI: 0.93–1.10) were not significantly reduced [30]. That meta-analysis also reported a smaller reduction in all-cause mortality (HR = 0.76, 95% CI: 0.66–0.87) than observed in our cohort (HR = 0.57), suggesting that our all-cause mortality estimate may be partly inflated by residual confounding or healthy-vaccinee bias. The large reduction in cancer deaths is unlikely to be directly attributable to PPSV23 and raises concern about residual healthy-vaccinee bias or unmeasured confounding. The reduction in external causes further supports this possibility. Therefore, pneumonia- and COPD-related mortality should be regarded as more specific endpoints, whereas the all-cause mortality association should be interpreted as supportive rather than definitive.

A notable contribution is the evaluation of COPD-related mortality, an endpoint seldom assessed in prior PPSV23 effectiveness research. The observed 33.5% reduction in COPD-related mortality is biologically plausible: COPD is characterized by impaired mucociliary clearance, chronic airway inflammation, and heightened susceptibility to severe infectious exacerbations, all of which can be triggered by pneumococcal infection [1,28].

Subgroup analyses revealed heterogeneous effects for COPD-related mortality. Protection was significant among males (VE = 42.3%) but not among females, and increased with age: VE was 45.6% in adults aged 70–79 years and 52.0% in those aged ≥80 years, whereas the effect did not reach statistical significance in the 60–69 years age group. This age gradient aligns with the 82.9-year life expectancy of Guangzhou residents: adults aged ≥70 years are closer to the end of the life course, with higher baseline mortality, more comorbidities, and greater frailty [31]. Thus, although the relative effectiveness of PPSV23 against all-cause and pneumonia-related mortality was broadly consistent across age, sex, and district strata (with no significant interactions), vaccination delivers greater absolute mortality benefits in older age groups. This finding supports the prioritization of adults aged ≥65 years in public health immunization programs, as they derive the largest clinical and population-level benefit.

Subgroup analyses showed influenza vaccination augmented PPSV23 efficacy: VE against pneumonia-related mortality and COPD-related mortality was 48.0% and 38.2% in co-vaccinated adults, respectively. In unvaccinated adults, significant protection persisted only for all-cause mortality (VE = 41.3%). This aligns with synergistic pathogenesis of influenza and pneumococcus [32,33,34], supporting combined vaccination strategies [35]. A divergent point estimate was noted in analyses of COPD-related mortality among individuals without influenza vaccination: VE was −95.6%, which, although not statistically significant, was directionally opposite. This finding may suggest residual confounding or differences in baseline health status—individuals not receiving influenza vaccine may be in poorer health, potentially masking the protective effect of PPSV23. This observation warrants further investigation.

Antimicrobial resistance is another important consideration in pneumococcal disease among older adults. Vaccination is increasingly recognized as a complementary tool to address antimicrobial resistance: pneumococcal vaccines protect against infection with vaccine serotypes independently of the antibiotic susceptibility of the infecting strain, and reductions in antibiotic use and antibiotic-resistant invasive pneumococcal disease have been most clearly demonstrated following pneumococcal conjugate vaccine introduction [36]. This is particularly relevant in China, where national surveillance data from elderly patients show macrolide (erythromycin) resistance in approximately 93% of Streptococcus pneumoniae isolates [37], reinforcing the public-health value of PPSV23 vaccination among older adults in China.

Overall, our findings suggest that PPSV23 vaccination is associated with reduced mortality among older adults in Guangzhou, with important implications for China. Although higher-valent conjugate vaccines are increasingly adopted globally, PPSV23 remains central to pneumococcal prevention among Chinese adults, given its reliable domestic supply and continued inclusion in routine prevention and control strategies in public health programs across China [38]. Notably, immunogenicity does not equate to durable clinical protection. Given the low and uneven pneumococcal vaccination coverage among Chinese adults [39,40,41,42], our results support expanding coverage, particularly in the oldest-old and those with chronic respiratory diseases. Our results are generalizable to Chinese clinical practice, as most participants received domestic PPSV23, but are limited to urban settings with mature surveillance. Key strengths include a large population, linkage of immunization and mortality registries across all 11 Guangzhou districts, propensity score matching, Cox proportional hazards modeling, evaluation of three mortality endpoints, and the use of a negative control outcome with E-value sensitivity analysis.

Limitations warrant consideration. First, healthy-vaccinee bias remains the primary threat to validity despite propensity score matching, multivariable adjustment, and E-value assessment. Vaccinated older adults tend to have better health awareness, higher socioeconomic status, and fewer comorbidities. Although matching balanced observed covariates, we lacked data on smoking, disease severity (cardiovascular disease, diabetes, immunocompromising conditions), activities of daily living, and prior hospitalization. Residual confounding from these unmeasured factors may overestimate vaccine effectiveness, particularly for all-cause mortality. In particular, the all-cause mortality association may be more vulnerable to residual healthy-vaccinee bias and unmeasured confounding than the pneumonia- and COPD-specific outcomes; Studies incorporating these variables are needed to validate these findings. Second, reliance on Mortality Surveillance Systems may involve underreporting or cause-of-death misclassification, affecting estimate precision. In addition, it does not record the setting of infection acquisition for individual cases (community-acquired vs. hospital-acquired/healthcare-associated), nor does it contain microbiological isolates or antimicrobial susceptibility data. Consequently, deaths from hospital-acquired or healthcare-associated pneumococcal infection could not be analyzed as a separate outcome, and our estimates primarily reflect community-acquired pneumococcal disease. Third, our study was restricted to adults aged ≥60 years, and findings may not be generalizable to younger age groups. Future research should extend beyond the ≥60-year-old population to evaluate the value of earlier PPSV23 vaccination among middle-aged adults with chronic underlying conditions, particularly as population life expectancy continues to increase. Studies linking immunization and mortality registries with comprehensive clinical data are also warranted to quantify absolute benefits in high-risk populations (e.g., patients hospitalized for COPD), and directly compare PPSV23-based strategies with sequential or higher-valent conjugate vaccines in real-world Chinese settings.

## 5. Conclusions

PPSV23 vaccination is associated with meaningful mortality reductions in older adults in Guangzhou, with more specific evidence for pneumonia- and COPD-related death. The all-cause mortality association should be interpreted cautiously because of possible residual confounding. These findings support PPSV23’s role in healthy-aging immunization programs in China and highlight the value of combined pneumococcal and influenza vaccination.

## Figures and Tables

**Table 1:** Baseline Characteristics of Adults Aged ≥60 Years in Guangzhou by PPSV23 Vaccination Status, Before and After Propensity Score Matching.

Variable	Before Propensity Score Matching: PPSV23 Vaccination Status				After Propensity Score Matching: PPSV23 Vaccination Status			
	Total (*n* = 653,414)	Unvaccinated Group (*n* = 372,583)	Vaccinated Group (*n* = 280,831)	SMD	Total (*n* = 453,500)	Unvaccinated Group (*n* = 226,750)	Vaccinated Group (*n* = 226,750)	SMD
Age at Enrollment (mean ± SD)	68.87 ± 6.12	69.14 ± 6.39	68.52 ± 5.71	0.102	68.53 ± 5.81	68.51 ± 5.91	68.56 ± 5.70	0.009
Sex = Male (%)	292,077 (44.70)	169,479 (45.49)	122,598 (43.66)	0.037	192,050 (42.35)	99,395 (43.83)	92,655 (40.86)	0.06
Regional Distribution = Central Urban Areas (%)	284,962 (43.61)	193,434 (51.92)	91,528 (32.59)	0.399	175,437 (38.69)	86,714 (38.24)	88,723 (39.13)	0.018
Urbanization Level = Rural Township (%)	177,471 (27.16)	80,495 (21.60)	96,976 (34.53)	0.291	147,136 (32.44)	64,853 (28.60)	82,283 (36.29)	0.165
Influenza Vaccine Doses During Study Period (mean ± SD)	1.10 ± 0.86	1.09 ± 0.74	1.12 ± 0.99	0.043	1.12 ± 0.91	1.12 ± 0.89	1.11 ± 0.92	0.018
Influenza Vaccination = Yes (%)	490,847 (75.12)	301,011 (80.79)	189,836 (67.60)	0.305	327,745 (72.27)	166,091 (73.25)	161,654 (71.29)	0.044
Mortality Status (%)				0.195				0.164
Alive	623,989 (95.50)	349,570 (93.82)	274,419 (97.72)		436,383 (96.23)	214,666 (94.67)	221,717 (97.78)	
All-Cause Mortality	29,425 (4.50)	23,013 (6.18)	6412 (2.28)		17,117 (3.77)	12,084 (5.33)	5033 (2.22)	
Circulatory System Diseases	11,434 (1.75)	8895 (2.39)	2539 (0.90)		6581 (1.45)	4588 (2.02)	1993 (0.88)	
Coronary heart disease	5146 (0.79)	4002 (1.07)	1144 (0.41)		2927 (0.65)	2020 (0.89)	907 (0.40)	
Cerebrovascular disease	4333 (0.66)	3329 (0.89)	1004 (0.36)		2574 (0.57)	1797 (0.79)	777 (0.34)	
Malignant Tumors	8649 (1.32)	6724 (1.80)	1925 (0.69)		5290 (1.17)	3768 (1.66)	1522 (0.67)	
Digestive system tumors	3237 (0.50)	2521 (0.68)	716 (0.25)		1408 (0.62)	566 (0.25)	1974 (0.44)	
Respiratory system tumors	2846 (0.44)	2204 (0.59)	642 (0.23)		1205 (0.53)	505 (0.22)	1710 (0.38)	
Respiratory System Diseases	5081 (0.78)	4054 (1.09)	1027 (0.37)		2740 (0.60)	1920 (0.85)	820 (0.36)	
External Causes	1427 (0.22)	1099 (0.29)	328 (0.12)		878 (0.19)	635 (0.28)	243 (0.11)	
Digestive System Diseases	891 (0.14)	668 (0.18)	223 (0.08)		526 (0.12)	361 (0.16)	165 (0.07)	
Endocrine, Nutritional and Metabolic Diseases	812 (0.12)	666 (0.18)	146 (0.05)		476 (0.10)	358 (0.16)	118 (0.05)	
Others Causes	351 (0.05)	279 (0.07)	72 (0.03)		195 (0.04)	138 (0.06)	57 (0.03)	
Genitourinary System Diseases	301 (0.05)	239 (0.06)	62 (0.02)		173 (0.04)	124 (0.05)	49 (0.02)	
Infectious Diseases	271 (0.04)	215 (0.06)	56 (0.02)		151 (0.03)	110 (0.05)	41 (0.02)	
Nervous System Diseases	208 (0.03)	174 (0.05)	34 (0.01)		107 (0.02)	82 (0.04)	25 (0.01)	
Follow-up Duration (Years, mean ± SD)	5.59 ± 0.41	5.55 ± 0.51	5.64 ± 0.18	0.233	5.61 ± 0.35	5.57 ± 0.46	5.64 ± 0.18	0.203

**Table 2:** Association of PPSV23 Vaccination With Mortality: Time-Dependent Cox Regression Models.

Outcome Variable #	PPSV23-Vaccinated Group (*n* = 226,750)			Unvaccinated Group (*n* = 226,750)			Univariate Model	Multivariate Model				
	Number of Deaths	Person-Years of Observation	Mortality Density	Number of Deaths	Person-Years of Observation	Mortality Density	Crude HR	Adj HR	VE(%)	*p* Value	E-Value	E-Value for Lower CI of HR
			(95% CI)			(95% CI)	(95% CI)	(95% CI)	(95%C)		For HR	
All-Cause Mortality	5033	1,279,358	3.93	12,084	1,263,116	9.57	0.67	0.57	43.1	<0.001	2.31	2.24
			(3.83, 4.04)			(9.40, 9.74)	(0.66–0.69)	(0.55–0.59)	(41.2, 45.0)			
Pneumonia-Related Mortality	432	1,279,358	0.34	1174	1,263,116	0.93	0.67	0.54	46.2	<0.001	2.44	2.18
			(0.31, 0.37)			(0.88, 0.98)	(0.61–0.74)	(0.48–0.60)	(39.6, 52.1)			
COPD-Related Mortality	281	1,279,358	0.22	587	1,263,116	0.46	0.79	0.66	33.5	<0.001	1.98	1.63
			(0.19, 0.25)			(0.43, 0.50)	(0.71–0.89)	(0.56–0.79)	(20.9, 44.2)			

#: Propensity-score-matched analysis. Adj HR: adjusted for age, sex, regional distribution, urbanization level and influenza vaccination. E-value: minimum RR an unmeasured confounder would need with both exposure and outcome to fully explain the observed association (Adj HR = 1.0). E-value for lower CI: minimum confounding strength to shift the 95% CI lower bound to 1.0.

**Table 3:** Association of PPSV23 Vaccination With Mortality Across Subgroups: Time-Dependent Cox Regression Models.

Outcome Variable #	Stratifying Variable	Number of Deaths/Person-Years (Mortality Density)		Adj HR (95% CI)	VE(%) (95% CI,%)	*p* Value	*p* for Interaction
		Vaccinated Group	Unvaccinated Group				
All-cause Mortality	Gender						0.089
	Female	1979/757,612 (2.61)	4853/713,027 (6.81)	0.52 (0.49–0.56)	47.6 (44.2, 50.9)	<0.001	
	Male	3054/521,746 (5.85)	7231/550,090 (13.15)	0.59 (0.56–0.62)	41.4 (38.4, 44.3)	<0.001	
	Age						0.991
	60–69 years	1677/850,195 (1.97)	4150/850,540 (4.88)	0.56 (0.52–0.59)	44.3 (40.7, 47.6)	<0.001	
	70–79 years	2111/363,753 (5.80)	5102/346,635 (14.72)	0.51 (0.48–0.55)	49.0 (45.4, 52.2)	<0.001	
	≥80 years	1245/65,411 (19.03)	2832/65,941 (42.95)	0.60 (0.54–0.68)	39.5 (32.1, 46.2)	<0.001	
	Regional Distribution						0.098
	Non-central urban area	3355/778,611 (4.31)	8544/777,909 (10.98)	0.56 (0.54–0.59)	44.0 (41.4, 46.5)	<0.001	
	Central urban area	1678/500,747 (3.35)	3540/485,207 (7.30)	0.59 (0.56–0.63)	40.9 (37.3, 44.3)	<0.001	
	Urbanization level						0.47
	Township	2208/464,092 (4.76)	4908/358,517 (13.69)	0.51 (0.48–0.55)	49.0 (45.4, 52.4)	<0.001	
	Subdistrict	2825/815,266 (3.47)	7176/904,599 (7.93)	0.57 (0.55–0.60)	42.7 (40.2, 45.1)	<0.001	
	Influenza Vaccine						0.789
	Unvaccinated	1762/367,049 (4.80)	2106/339,725 (6.20)	0.59 (0.51–0.67)	41.3 (32.8, 48.7)	<0.001	
	Vaccinated	3271/912,309 (3.59)	9978/923,391 (10.81)	0.56 (0.53–0.58)	44.4 (42.2, 46.6)	<0.001	
Pneumonia-related Mortality	Gender						0.2
	Female	156/757,612 (0.21)	428/713,027 (0.60)	0.60 (0.48–0.75)	39.9 (25.4, 51.6)	<0.001	
	Male	276/521,746 (0.53)	746/550,090 (1.36)	0.51 (0.43–0.60)	49.3 (40.0, 57.1)	<0.001	
	Age						0.243
	60–69 years	106/850,195 (0.12)	253/850,540 (0.30)	0.59 (0.46–0.75)	40.9 (24.5, 53.8)	<0.001	
	70–79 years	156/363,753 (0.43)	492/346,635 (1.42)	0.51 (0.42–0.63)	48.7 (37.2, 58.2)	<0.001	
	≥80 years	170/65,411 (2.60)	429/65,941 (6.51)	0.48 (0.36–0.63)	52.3 (37.4, 63.7)	<0.001	
	Regional Distribution						0.937
	Non-central urban area	174/778,611 (0.22)	564/777,909 (0.73)	0.56 (0.45–0.69)	44.1 (31.3, 54.5)	<0.001	
	Central urban area	258/500,747 (0.52)	610/485,207 (1.26)	0.52 (0.44–0.62)	47.7 (38.4, 55.6)	<0.001	
	Urbanization level						0.561
	Township	90/464,092 (0.19)	268/358,517 (0.75)	0.42 (0.30–0.60)	57.6 (40.0, 70.1)	<0.001	
	Subdistrict	342/815,266 (0.42)	906/904,599 (1.00)	0.54 (0.47–0.62)	45.7 (37.8, 52.5)	<0.001	
	Influenza Vaccine						0.767
	Unvaccinated	127/367,049 (0.35)	166/339,725 (0.49)	0.69 (0.44–1.07)	31.2 (−7.0, 55.8)	0.097	
	Vaccinated	305/912,309 (0.33)	1008/923,391 (1.09)	0.52 (0.46–0.59)	48.0 (40.7, 54.5)	<0.001	
COPD-related Mortality	Gender						0.007
	Female	42/757,612 (0.06)	91/713,027 (0.13)	0.80 (0.39–1.64)	20.2 (−63.8, 61.1)	0.539	
	Male	239/521,746 (0.46)	496/550,090 (0.90)	0.58 (0.47–0.71)	42.3 (28.7, 53.3)	<0.001	
	Age						0.023
	60–69 years	60/850,195 (0.07)	112/850,540 (0.13)	0.71 (0.50–1.01)	28.8 (−1.3, 50.0)	0.059	
	70–79 years	139/363,753 (0.38)	276/346,635 (0.80)	0.54 (0.37–0.81)	45.6 (19.0, 63.5)	0.003	
	≥80 years	82/65,411 (1.25)	199/65,941 (3.02)	0.48 (0.29–0.78)	52.0 (21.6, 70.7)	0.003	
	Regional Distribution						0.449
	Non-central urban area	203/778,611 (0.26)	459/777,909 (0.59)	0.57 (0.45–0.72)	42.9 (28.3, 54.5)	<0.001	
	Central urban area	78/500,747 (0.16)	128/485,207 (0.26)	0.72 (0.51–1.01)	27.9 (−1.5, 48.8)	0.061	
	Urbanization level						0.064
	Township	133/464,092 (0.29)	278/358,517 (0.78)	0.48 (0.33–0.69)	52.2 (30.7, 67)	<0.001	
	Subdistrict	148/815,266 (0.18)	309/904,599 (0.34)	0.75 (0.60–0.94)	24.7 (5.6, 40.0)	0.014	
	Influenza Vaccine						0.149
	Unvaccinated	110/367,049 (0.30)	69/339,725 (0.20)	1.96 (0.46–8.25)	−95.6 (−724.8, 53.6)	0.361	
	Vaccinated	171/912,309 (0.19)	518/923,391 (0.56)	0.62 (0.50–0.76)	38.2 (24.1, 49.7)	<0.001	

#: Propensity-score-matched analysis. Adj HR: Adjusted for age, sex, regional distribution, urbanization level, and influenza vaccination.

**Table 4:** Association of PPSV23 Vaccination With Traffic Accident Mortality: Time-Dependent Cox Regression Models.

Outcome Variable #	Vaccinated Group (*n* = 226,750)			Unvaccinated Group (*n* = 226,750)			Crude Model	Adjusted Model				
	Deaths Number	Observed Person-Years	Mortality Density (95% CI)	Deaths Number	Observed Person-Years	Mortality Density (95% CI)	Crude HR (95% CI)	Adj HR (95% CI)	VE(%) (95% CI)	*p* Value	E-Value For HR	E-Value for Lower CI of HR
Traffic Accidents Mortality	50	1,279,358	0.04 (0.03, 0.05)	125	1,263,116	0.10 (0.08, 0.12)	0.83 (0.62–1.10)	0.79 (0.53–1.18)	21.1 (−17.8, 47.1)	0.247	1.64	1

#: Propensity-score-matched analysis. Adj HR: Adjusted for age, sex, regional distribution, urbanization level and influenza vaccination. E-value: Minimum RR an unmeasured confounder would need with both exposure and outcome to fully explain the observed association (Adj HR = 1.0). E-value for lower CI: Minimum confounding strength to shift the 95% CI lower bound to 1.

## Data Availability

Data cannot be shared openly due to the protection of patient information and privacy. Access to the data is subject to approval and a data-sharing agreement with the Guangzhou CDC.

## Abbreviations

The following abbreviations are used in this manuscript:

PPSV23	The 23-valent pneumococcal polysaccharide vaccine
Spn	Streptococcus pneumoniae
COPD	Chronic obstructive pulmonary disease
CAP	Community-acquired pneumonia
PCV13	The 13-valent pneumococcal conjugate vaccine
PSM	Propensity score matching
HR	Hazard ratio
VE	Vaccine effectiveness
